# The Sea Lamprey (*Petromyzon marinus*) Invasion: The Construction of an Invasive Animal Threatening a “Healthy” Great Lakes Ecosystem

**DOI:** 10.1093/jhmas/jrae046

**Published:** 2025-01-31

**Authors:** Vincent Bijman

**Affiliations:** Maastricht University, The Netherlands

**Keywords:** invasive species, history of problem animals, Great Lakes history, history of ecology, construction of environmental problems

## Abstract

During the late 1930s, Great Lakes fishermen became concerned because of the new occurrence of the sea lamprey (*Petromyzon marinus*). Originally an Atlantic coastal fish, it was allowed to migrate throughout the Great Lakes due to various canal extensions. By drawing from literature on the sociology of environmental problems and animal invasions, this article traces how the sea lamprey became problematized as a threatening invader between the late 1930s and early 1970s. Throughout this period, a broad coalition of fishery biologists, fishermen, politicians, and journalists were involved in framing the problem. Although sea lamprey research, localized control practices, and environmental discourses considerably changed, the sea lamprey continued to be regarded as an invasive fish that was not allowed to exist in the Great Lakes. The case shows how these shifting ways of understanding the problem in fact led to the continuation of past management directions.

During the late 1930s, many American and Canadian fishermen around the shores of the Great Lakes became worried following the news of a newly arrived predatory fish: the sea lamprey (*Petromyzon marinus*). The sea lamprey had traditionally not occurred in Lake Erie, Lake Huron, Lake Michigan, or Lake Superior, but only on the Atlantic east coast. However, individual sea lampreys were now more commonly observed. Firstly, they appeared in shallow and bright freshwater streams connected to the Great Lakes during sea lamprey spawning. Secondly, they were noted as physically attached to freshwater fish that were caught in high numbers by commercial fishermen such as lake trout (*Salvelinus namaycush*) and lake whitefish (*Coregonus clupeaformis*). Also, sea lamprey bite wounds were spotted on the sides of these caught fish, which made the fish unsellable.

The sudden arrival of the sea lamprey in the Great Lakes was an unintended consequence of canal constructions and extensions at Lake Ontario and Lake Erie, aimed at creating a continuous waterway in the world’s largest freshwater system. The Erie Canal was completed in 1825, and this large-scale water engineering project connected Lake Erie and the St. Lawrence River, allowing for inland shipping between the American east coast and Lake Erie. Moreover, the construction of the Welland Canal between Lake Ontario and Lake Erie bypassed Niagara Falls, which until then formed a natural barrier for the sea lamprey.^1^ The Welland Canal modifications finalized in 1919 also came with an alteration in waterflow, now moving unidirectionally from Lake Ontario to Lake Erie, allowing the sea lamprey passage to the other Great Lakes.^2^ Sea lampreys are anadromous – they migrate from the ocean to freshwater tributaries to spawn, and a population can also adapt to a full freshwater life. The sea lamprey utilized the new waterway from Lake Ontario into Lake Erie, perhaps some of them even utilizing their round suction-disc mouths to latch on to inland ships, and moved and expanded their population throughout the Great Lakes.^3^

Reports on the occurrence of attacks and subsequent fishery decline, made by commercial fishermen and confirmed by Great Lakes fishery biologists, were seen as proof of an overabundant sea lamprey population as the foremost pressing issue in Great Lakes fishery management. In contrast to typical predatory fish behavior, the sea lamprey, a fish that resembles an eel, does not attack and devour smaller fish, but instead utilizes a round suction cup mouth to latch on to prey fish, rasp a hole in their scales and feed on blood and body tissue, commonly defined as parasitization.^4^ In addition, fishermen noticed that the sea lamprey could not be utilized in the fishery industry in a similar manner to other freshwater fish and was therefore deemed worthless.^5^ Since the mid 1940s, the novel sea lamprey occurrence developed into a major invasive fish issue, in which this animal was targeted for extensive scientific studies in service of international control in American and Canadian waters.

This article explores the history of the sea lamprey invasion as a case study to understand how an animal that moves into a new environment becomes problematized and regarded as an “invader” affecting a “healthy environment” in times of changing perceptions of nature conservation and fishery management practices. These problematizations concern the ways in which a newly occurring animal species is framed, studied, and managed as a problem, invasive animal. These constructed representations are not static, but rather malleable and change over time. It is in fact this ongoing process of renewed problematization that contributes to the issue being perceived as still relevant today.

Concentrating on health in the history of animal invasions allows historians to tease out an important aspect of how invasions are framed. In the case of the sea lamprey in the period between 1930 and 1960, the most dominant discourse was one of utility, in which the sea lamprey was framed as a threat to the Great Lakes fishery resource by Great Lakes scientists, managers, and the media. However, throughout this period, the sea lamprey was increasingly framed as a health threat.

In the first decades after its introduction, the sea lamprey was presented by fishermen and Great Lakes fishery managers as a major threat to Great Lakes fisheries. Firstly, it was framed as a threat to healthy individual lake trout that constituted an important part of fishery revenues, and secondly as a threat to a healthy freshwater fishery industry as a whole. By the end of the 1960s it became newly problematized by a new generation of Great Lakes scientists as a threat to the Great Lakes as a healthy natural environment, presenting the sea lamprey as a non-native invader with a more systemic impact. This occurred in a transitional period for Great Lakes scientists and fishery managers, in which new Great Lakes management strategies were negotiated and introduced. While the Great Lakes became a topic in a national debate on environmental pollution, the sea lamprey invasion was renegotiated and found a new place as a part of a multivariate of issues that threatened an ailing Great Lakes ecosystem. This paper thus provides a novel insight into how the impact of the sea lamprey was perceived, valued, and changed, and how health gained a prominent role in mid twentieth-century invasive species history.

Although scientific studies, managerial activities, and environmental discourse regarding the Great Lakes as a natural space and its inhabiting wildlife changed considerably during the twentieth century, the sea lamprey continued to be regarded as a problematic invader that should be eradicated. This paradox shows how changes in environmental problematization did not necessarily lead to a reconsideration of a past environmental issue, but instead allows for the consolidation of the putative problem and the continuation of past managerial decisions. In fact, I argue that from the late 1960s onwards new ways of understanding the sea lamprey allowed for the adaptation of animal invasions to a novel environmentalist discourse, while past rhetoric and control practices continued to prevail. In this case this meant the continuation and expansion of existing managerial directions, with chemical control used as the primary method for Great Lakes-wide sea lamprey eradication.

The sea lamprey is an exceptional case in terms of periodization and scope. It became understood by a network of diverse human actors, including Great Lakes fishery scientists, fishermen, fishing industry representatives, and politicians as a threat to the fishery industry as early as the late-1930s. It continues to be a major Great Lakes fishery issue today. As a consequence of the case, an elaborate sea lamprey control network emerged, and international institutional integration was achieved in the form of the foundation of the Great Lakes Fisheries Commission (GLFC) as a bilateral authority for Great Lakes fishery science and management, following the signing of the 1954 Convention on Great Lakes Fisheries between the United States and Canada.^6^ Around the time of the signature, invasion ecology emerged as an ecological subdiscipline. Major cases such as the sea lamprey formed the empirical evidence of a new understanding of animal and plant invasions, as developed by ecologists such as Charles Elton.^7^

The history of the sea lamprey invasion has received attention by environmental historians of the Great Lakes, biologists with an interest in the history of sea lamprey control, and legal and environmental policy scholars studying the history of Great Lakes treaties and conventions.^8^ In particular the Great Lakes biologist Cory Brant has published a detailed monograph that discusses the history of the sea lamprey invasion and subsequent control efforts. Most of these publications follow a narrative that understands the sea lamprey as a damaging ecological invasion that affected Great Lakes economies and ecosystems, but that became successfully controlled after continuous research and control experiments.

In contrast to the current historical literature on the history of the sea lamprey invasion, in this article I follow a cultural historical approach to understand the ways in which the sea lamprey became problematized and considered a threat to a “healthy” Great Lakes environment. For this purpose, I combine two strands of theoretical literature. Firstly, the case is connected to the sociology of environmental problems. Since the 1970s, sociological interest emerged regarding the question of how social, and subsequently how environmental problems are constructed.^9^ In *Environmental Sociology,* John Hannigan argues that the construction of environmental problems requires a cast of involved social actors who are engaged in a “claims making process” that contributes to the construction of the environmental problem: the issue is defined, relevant claims are substantiated, and a course of managerial action is justified, in an attempt to remedy the putative problem.^10^

Secondly, I build on the work of animal historians and human-animal studies scholars who have recently developed an interest in how certain animals became identified, framed, and managed as problematic animals, such as invasives, pests, or vermin and perceived as affecting public health, economic production, or nature.^11^ In different times and places, animals such as rats, badgers, and sparrows became subjected to elaborate research and state supported control programs. In recent publications on animal pests as vectors for transmitted diseases, scholars have unpacked the ways in which pest animals have come to function as scapegoats for complex public health problems.^12^ As the case of the sea lamprey shows, this also extends to healthy industries and environmental health. The sea lamprey was scapegoated and became a means to divert from broader and more complex environmental issues, in particular fishery decline due to overfishing and lake pollution.^13^

In contrast to pest animals, animal invasions constitute a particular category in which the movement to a new space is inherent to the way in which the problem is perceived. Until recently, historians interested in the history of animal invasions have emphasized continuities of how invasions have been conceptualized in in the late nineteenth century as imperial processes.^14^ Although these continuities are important to the ways in which animal invasions have been perceived throughout the twentieth century, this article follows previous publications that shift the focus to the mid-twentieth century, to allow a better understanding of how earlier representations of invasions have been reconceptualized to remain relevant.^15^

By following these two strands of literature on the sociology of environmental problems and the animal history of pests and invasions, the sea lamprey case study can help understand how animal agency in human-animal interactions shapes the way that animal related environmental issues are problematized. In turn, the sociology of environmental problems provides new insights into the construction of the animal invasion.

This article is divided into two sections, following the chronology of sea lamprey science, management, and media. The introduction of a novel control technique in the form of 3-trifluormethyl-4-nitrophenol (TFM or commonly “lampricide”), a toxin that was perceived to specifically target ammocetes (sea lamprey larvae) is presented as a caesura, dividing the two parts. This moment indeed meant a turning point in sea lamprey science and control while simultaneously the representation of the problem only changed gradually in the following years. The emergence of a new environmental movement in the early 1960s led to broader public concern for pollution affecting the Great Lakes as a healthy ecosystem and related ecosystem services.

## The Representation of the Sea Lamprey as an Alien Threat to Great Lakes Fisheries and US Food Security

Humans and lampreys have a history that spans centuries, in which, depending on location and context, lampreys became regarded as an integral part of nature, valued food source, prized delicacy, or problematic pest. In medieval Britain, the delicacy of a lamprey stew was a household dish of the English royal family, especially enjoyed by Henry I. According to one legend, overeating this stew led to the early death of the king, although this story has been recently disputed.^16^ In the US, pacific lampreys (*Entosphenus tridentatus*) have been hunted seasonally by the members of local indigenous tribes for centuries, and are now regarded by conservationists as in danger of extinction and are therefore subjected to conservation measures.^17^

However, in contrast to the long-standing human interest in lampreys, scientific studies have lagged behind. After its classification by Carl Linnaeus in 1758, the sea lamprey received only sporadic attention from naturalists in the US, with only a moderate increase in interest during the late-nineteenth century.^18^ The few naturalists who studied the sea lamprey focused on the individuals that had found a new home in the Finger Lakes of New York State, near Lake Ontario. One of these scholars, H.A. Surface, already emphasized the predatorial behavior of the fish, and recognized no human or natural benefit in the animal. He feared the potential expansion of the sea lamprey into freshwater, writing in 1897 that from “every economical standpoint it would appear to be advantageous to rid the world entirely of the lampreys.”^19^

## A Novel Fish is Turned into a Problematic Invader

The process of problematizing the sea lamprey as a threatening invader started quickly after the establishment of a stable sea lamprey population in Lake Huron in the early 1930s.^20^ The first concerned reports were issued by fishermen who resided in local fishing towns such as Rogers City, Michigan. In an article titled “Dread Sea Lampreys in First Spawn Run,” published on 15 August 1937 in the *Milwaukee Journal*, Clarence Mertz, a Rogers City commercial fisherman, was quoted to have said that the catch of lake trout in Hammond Bay had dropped to “practically nothing” during the month of June.^21^ According to Mertz, “lamprey scars were found on four out of every five lake trout caught in the bay.” The author of the article further noted that “the lamprey attaches itself to its victim by means of a sucking mouth. It rasps a hole through the skin of the fish and gorges itself on its victim’s blood.”^22^

The emphasis on scarring or damaging lake trout alluded to deviance in contrast to a previous healthy condition of fish populations that the sea lamprey was allegedly threatening. The Hammond Bay and the nearby Ocqueoc River would become the primary location for early sea lamprey spawning stream studies for Great Lakes wide control, and an abandoned coast guard house at Hammond Bay was acquired by sea lamprey control officers in the early 1950s to establish a sea lamprey field laboratory for elaborate control studies.^23^

Great lakes fishing yields had been fluctuating for decades by the 1930s, and fishermen were anxious about another setback, while commercial market demands required continuous high fishing yields to maintain a profitable business.^24^ Fishermen were not, however, the only human actors involved. While they had a major role in identifying the problem, Great Lakes fishery biologists took the lead in defining and suggesting policy directions. One of the earliest reports was provided by Carl Leavitt Hubbs, ichthyologist at the University of Michigan, and T.E.B. Pope, curator of lower zoology at the Milwaukee Public Museum, who reported on the first noted sea lamprey spawning run in Lake Erie in 1932. The pair discussed the sea lamprey in “The Spread of the Sea Lamprey Through the Great Lakes” as follows: “This eel-like creature, averaging about 15 inches when mature, clings to the larger food fishes with a round sucker-mouth, beset with rows of strong, horny teeth; then rasps open a hole in the skin of its victim by means of its serrated tongue plates, and injects an anticoagulating substance into the wound, to insure the free flow of the victim’s blood, with which the parasite gorges itself.”^25^ The descriptions by Mertz, Hubbs, and Pope illustrate two important aspects of the way in which the sea lamprey was initially problematized. Firstly, they drew the causal connection between increasing sea lamprey numbers and predation to declining lake trout catches. Secondly they established the idea that the sea lamprey was a unique fish, with a particular snake- or eel-like appearance and parasitic predatory behavior, threatening the health of valued fish species.

Since the occurrence of the sea lamprey in Lake Erie and its subsequent move to Lake Huron in the 1930s, the species was closely observed by John Van Oosten, a Great Lakes biologist who came to prominence as an authoritative voice in Great Lakes fishery conservation. At the time Van Oosten was the director of the US Bureau of Fisheries Great Lakes Laboratory in Ann Arbor, Michigan.^26^ He informed himself about the sea lamprey in conversation with local fishermen and had specimens sent to his office in Ann Arbor for analysis.

Since the late-nineteenth century, Great Lakes fisheries had been severely pressured by declining fishing yields due to overfishing and lake pollution. Important causes for pollution were the dumping of sawdust and other industrial wastes. Further human activities such as waterway engineering and land clearance for agricultural purposes also had a severe impact on the Great Lakes environment. Fishery biologists had been appointed to advise on conservation strategies for continuous fishery resource extraction. By the time Van Oosten started his work at the Great Lakes, the landlocked Atlantic salmon (*Salmo salar*) in Lake Ontario had already been fished to extinction.^27^ In political and legal terms, the Great Lakes area had been divided among eight American states and the Canadian province of Ontario – each with the mandate to organize fishery policy for their own jurisdiction. This made cohesive fishery management difficult and led to policy discussions on unfair competition with regions having different fishery regulations in terms of fish net mesh size, closed season fishing, and fishery quotas.^28^

In this complex political environment, Van Oosten, with only limited financial means and a few staff members, attempted to push for additional funds for fishery research, the collection of coherent fishery data, and integration of fishery regulation. He introduced “catch per unit of effort” as a primary statistic in Great Lakes fishery research. This allowed scientists to know how many fish were taken in comparison to the length and intensity of the fishing activity (for example the type of gear used and the duration of nets cast).^29^ Van Oosten also became one of the driving figures in the organization of a conservation alliance following the cisco crash in Lake Erie of 1925. The population of the cisco (*Coregonus artedi*), a salmonid fish that was severely overfished, experienced a sudden population collapse in 1925 and an interregional meeting of conservationists involved in Lake Erie fisheries was organized to discuss the matter.^30^ This network was maintained in the subsequent years and formed the basis of an alliance that eventually engaged with the sea lamprey issue.^31^ In addition, Van Oosten had been a member of an International Board of Inquiry established in 1938 that provided an extensive report on the status of Great Lakes fisheries with suggestions for fishery conservation. The board organized twenty-nine public hearings, and on 6 August 1942 they submitted a report that called for joint action of Great Lakes governments to organize conservation.^32^

**Figure 1.**
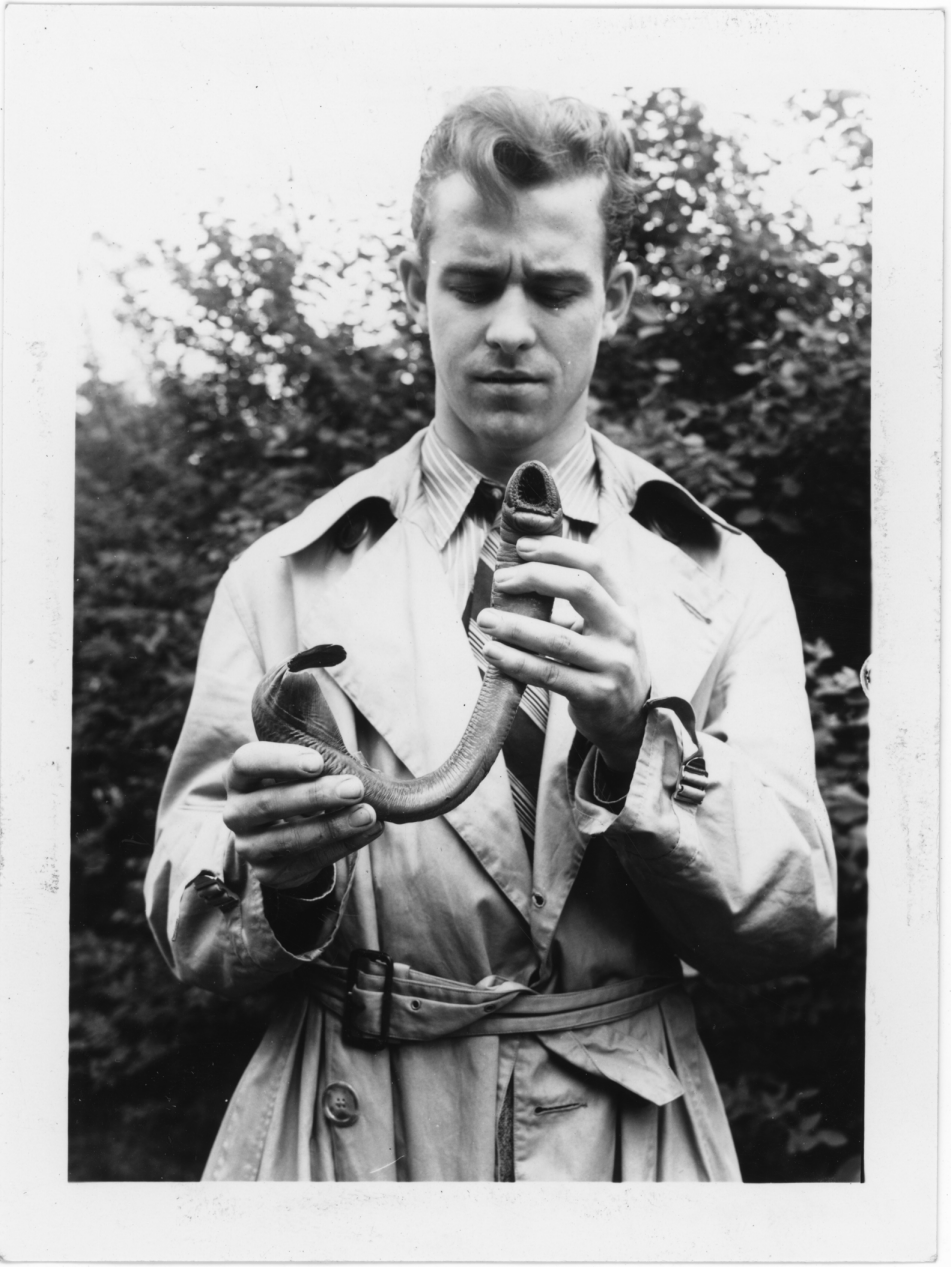
Man holding lamprey. Total length 453 mm (17.8 in.) Male. Dated 27 May 1939. Photo taken at Yates’s Water Wheel, Clinton River by H.J. Deason. By the courtesy of the US Geological Survey, Great Lakes Science Center, Ann Arbor, Michigan, 1939-A-1_a571.

Van Oosten organized the first sea lamprey research and control activities in the late 1930s, and learned about its migration, reproductive habits, and predatory activities (Figure 1). In addition, a first control experiment was executed by a group of concerned fishermen led by the Presque Isle County Sportsman’s Club, who went out to spear the lampreys in the Ocqueoc River and built a first provisional trapping weir. At the time, the Ocqueoc River in Michigan near Rogers City contained the only confirmed sea lamprey spawning location.^33^ This location proved not only to be important in the first identification of the problem, but also became one of the most important sites for sea lamprey research. However, wartime efforts caused the reduction of means for further sea lamprey research and control efforts, and work on the issue came to a standstill, while the numbers of lake trout caught by fishermen saw a sharp decline in the postwar years with only fifty percent of the averaged 6.6 million kilo per year yield and almost no return in Lake Michigan and Lake Huron by 1950. Lake Superior lake trout fishing continued until its full collapse in 1960.^34^

After the Second World War, the sea lamprey issue was pushed to the federal government by Van Oosten and his colleague Claude Ver Duin, representative of the Michigan Fish Producers Association and editor of *The Fisherman* newspaper, supported by senior staff members of the Fish and Wildlife Service.^35^ They argued for their case during a hearing of the Merchant Marine and Fisheries Committee for the House of Representatives, ominously titled *Menace of the Sea Lamprey*. During his statement, Ver Duin presented the sea lamprey as an excessive killer that would continue its predation after the valuable lake trout was wiped out by attacking other fish such as lake whitefish, white suckers (*Catostomus commersonii*), and bullhead catfish (*Ameiurus*), “so that no fish in the Great Lakes are safe from the attack of the lamprey” and the fishermen could not divert to fishing other species.^36^ In addition, Frederick Van Ness Bradley, a Republican representative from Michigan, emphasized that, “it is a national problem when they are driving our fishermen off the Great Lakes, […].” He added, “I think it is a menace that is a national problem, because, after all, people from all States of the Union eat our fish on the Great Lakes, and they are not going to continue to eat them very much longer if the eel keeps spreading year after year as it apparently has.”^37^ These discussions resulted in a house resolution (H.J.Res 366), that stated the sea lamprey is “increasing greatly in abundance” and is “causing injury to and destruction of the fishery resources of the Great Lakes” – “an indispensable and irreplaceable source of food” and gave authorization for research and control activities for “the elimination and eradication of the sea lamprey population of the Great Lakes” during a period of ten years.^38^

The committee debates and subsequent resolution marked the start of the representation of the sea lamprey invasion as a national food security issue. It provided Van Oosten and his colleagues with the required financial and political support to develop sea lamprey research and control activities to, if possible, fully eradicate the Great Lakes sea lamprey population. Van Oosten continued by co-organizing the first Great Lakes Sea Lamprey conference held in Ann Arbor on 14-15 November 1946 that further strengthened the ties within the sea lamprey research and control coalition and outlined the following steps in preparing for managerial activities.^39^ These included further investigations on the various phases of sea lamprey life history, surveying spawning streams to map sea lamprey presence across the Great Lakes, understanding the characteristics of the streams suitable for lamprey spawning, studying the movement and migration of sea lampreys, searching for natural enemies that could be released, studying the physiology of lampreys and understanding what intervention (poison, nets, weirs, electric, illuminating or electric devices) could be used in sea lamprey control.^40^

Subsequent hearings of the Committee on Merchant Marine and Fisheries of the House of Representatives in 1949 shed further light on the justification for sea lamprey control. Fred Westerman, chief of the Michigan Department of Conservation’s Fisheries Division for the US Fish and Wildlife Service between 1925 and 1959, stated in response to a question on whether other factors could have contributed to lake trout decline:

I would say, sir, that there is room for doubt there. But it looks as if this were the chief factor at least. We recognize that there are fluctuations in abundance in different species and in different localities and in different lakes. But this has been so widespread and so consistent, and so progressive along with the increase in numbers of the lamprey, and the feeling of the fishermen from so many different localities, that it looks as if that was the chief factor that is responsible for this great decline in lake trout. We never have experienced anything like it before.^41^

He also stated that there was no further research on other possible causes for lake trout decline. In addition, according to Claude Ver Duin, “the No. 1 problem at this time is the sea lamprey. All other problems are insignificant when compared with this one.”^42^ This monocausal justification has regularly been disputed since the late 1960s.^43^ More recently, environmental historian Nancy Langston argued that both overfishing and pollution led to habitat degradation and had already seriously affected the lake trout population.^44^

Around the late 1940s and with the establishment of federal support for sea lamprey research and control, the problem seemed clearly delineated. The occurrence of the sea lamprey, the observation of bite marks on lake trout and coinciding collapsing fish numbers were regarded as a sound justification for the claim that the sea lamprey was the most prominent cause for the calamitous state of Great Lakes fisheries. In committee hearings, scientific reports, and newspaper publications the sea lamprey was represented as an alien other that did not traditionally occur in the Great Lakes, and had an appearance and behavior that strongly deviated from what was commonly considered fish-like. In the 1949 hearing, Van Oosten doubted whether they could even be called a fish: “In Lake Superior we now have evidence of the adult fish – I call them fish, but we had better stick to the term sea lamprey, although we do call them fish – in five tributaries of Lake Superior[…].”^45^ Also a physical specimen even caused a certain sense of revulsion amongst the participants during a statement by Ver Duin: “It is a kind of a messy thing to pass around, but if you will pass around, and notice the mouth on it, it has a circular disk, with a lot of sharp teeth in it, and it burrows into the side of the fish and sucks the blood and then leaves the fish to die.” […] Mr. Allen intervened, “If I may interrupt, Mr. Chairman, no one wants this lamprey.” Mr. Ver Duin responded, “I assure you we don’t want it either.”^46^

The place of the first control attempts, the Ocqueoc River on the north-eastern shore of the lower peninsula of Michigan, was selected as the starting point for extensive sea lamprey life history studies and control trials. These studied were supervised by Vernon Applegate, a young PhD candidate at the University of Michigan who published his results in a dissertation titled *Natural History of the Sea Lamprey, Petromyzon Marinus, in Michigan* in 1950 after two years of springtime spawning stream fieldwork.^47^

Based on this research, a detailed understanding of specific sea lamprey characteristics was obtained. This dissertation focused on its migratory, predatory, and reproductive behavior, relevant information to understand the problem and organize control activities. This was summarized in the description of the sea lamprey life cycle: after spawning, sea lampreys live for multiple years burrowed in the bottom of shallow streams and survive as filter feeders. After a certain time, they continue to grow in length, form a round suction cup mouth and disperse downstream to one of the Great Lakes. There they predate on various middle or larger sized fish, by rasping a hole in the side of the fish, inserting a liquid that dissolves tissue, and feed on blood. After a few years in this predatory stage, the lampreys finish feeding, their liver stops functioning and their digestive tract dissolves. They move upstream in small streams to spawn, build a nest in the gravel while utilizing their mouths to move the rocks and reproduce. After reproduction that can produce on average 150,000 eggs, the parent lampreys die.^48^

After the series of committee hearings, the issue of integrating Great Lakes fishery management policy took an important step by the signing of the Convention on Great Lakes Fisheries between the US and Canada and subsequent formation of the Great Lakes Fishery Commission (GLFC) in 1954 that received the authority to coordinate Great Lakes fishery management.^49^ The most important foundational task of the GLFC was to take a leading role in the management of sea lamprey control. Simultaneously, mechanical control activities in the form of weir trapping and electro-fencing to remove adult sea lampreys were expanded across the Great Lakes during the 1950s, but these measures were a costly and time-consuming endeavor.

## The Sea Lamprey: Invader of the Great Lakes Ecosystem

When Van Oosten retired in 1950, James Moffett took over the role as the coordinator of the sea lamprey control program and shifted emphasis to toxin testing for the purpose of sea lamprey control. This happened in the context of the toxin craze in pest management: a rapid increase in use of pesticides as a promising solution for various pests. In particular, DDT became widely utilized.^50^ An abandoned coast guard station at Hammond Bay, near the mouth of the Ocqueoc River, was purchased as a field station for pesticide testing. Here various compounds were tested, and after eight years of arduous testing, in 1958 a toxin, 3-trifluoromethyl-4-nitrophenol (TFM) was selected that was perceived as highly effective to target sea lamprey ammocetes (sea lamprey juveniles in their larval state), while not harming other fish or the environment. Both sea lamprey ammocetes and a lake trout were placed in a water basin, and TFM was added. While the ammocetes died, the lake trout remained lively and seemingly unscathed.^51^ TFM was also a pesticide that could be mass produced; from 1958 it was added to spawning streams of sea lampreys.

During the time of testing TFM and its precursors, scientists discussed its effect on healthy test subjects to understand whether the toxin application would affect other fish, in particular lake trout.^52^ The consideration of health did at this time not yet commonly extend to the Great Lakes as a natural space, when the sea lamprey was discussed. Instead, the Great Lakes itself continued to be described by fisheries scientists, managers, and the media as a space for fishery resources. However, within a decade this perspective started to shift.

From the spring of 1962, it became clear to Great Lakes fishery biologists that continuous TFM application could lower the sea lamprey population by ninety percent.^53^ These TFM operations were therefore expanded in the following years. They were coordinated from US Fish and Wildlife Service biological stations in Ludington (established in 1956) and Marquette (established in 1956) in Michigan, while the Canadian counterparts of the Department of Fisheries and Oceans established in 1966 the Sea Lamprey Control Centre (SLCC) in Sault Saint Marie, Ontario.^54^ The sea lamprey control officers now had the task of continuously returning to major spawning streams for renewed treatment, turning the control strategy from mechanical control (killing adult upstream migrating lampreys) to chemical control, targeting the smaller filter feeding ammocetes. It was an arduous field operation: trucks with barrels of lampricide had to be brought to the shores of the stream, rubber hoses had to be installed, and the lampricide had to be applied carefully in specific concentrations, depending on factors such as the stream size and temperature.^55^ The application of too little of the toxin would not have the desired effect and multiple generations of burrowed ammocetes could potentially survive, while too much led to unwanted non-target fish kills. In addition, some of the older mechanical control operations continued but declined as the primary control method.

The success of TFM in reducing the sea lamprey in significant numbers marked the transformation of the sea lamprey issue into a case of continuous pest management. Although total eradication was unlikely to be obtained with TFM application, it did lower the population to such an extent that the GLFC scientists were confident in starting lake trout hatching programs, and subsequently considered the introduction of non-native fish such as chinook salmon (*Oncorhynchus tshawytscha*) and coho salmon (*Oncorhynchus kisutch*) as a means for improving sport fishing activities.^56^ Control practices were expanded during the 1960s and 1970s across the Great Lakes, and the GLFC lobbied for financial support to be able to apply the lampricide at all necessary spawning streams for the foreseeable future. While the sea lamprey population had been successfully suppressed, the issue now became regarded as an environmental risk. Loosening repeated control operations due to lacking political interest and insufficient funds could lead to a quick reoccurrence of a large sea lamprey population, undoing the various control efforts since the late 1940s.

Although initially sea lamprey control was justified to protect the Great Lakes fishery industry and accompanying livelihoods, effective sea lamprey control in the late 1950s with the application of TFM did not result in the return of commercial fisheries as the most prominent commercial enterprise on the Great Lakes. Instead, sport fishery and its accompanying tourism industry boomed, mobilizing the urban middle class population who hoped to enjoy fishing and the Great Lakes as a natural space.^57^ In 1967, thousands of sport fishing enthusiasts embarked for angling the newly introduced coho salmon in an episode that became known as the “coho craze”: hundreds of eager fishermen lined up on fishing boats to fish the newly introduced coho salmon. Indeed, although the sea lamprey continued to be regarded as a threatening invasive, the introduction of other species deemed valuable was supported.

## An Ecological Approach to Sea Lamprey Science

By the end of the 1960s, the sea lamprey invasion was reframed as an ecosystemic problem, marking the start of extensive research in sea lamprey ecology and new eradication strategies. An early example of a shifting perspective on the Great Lakes as an ecosystem is found in a GLFC technical report on “The Ecology and Management of the Walleye in Western Lake Erie,” published by Vernon C. Applegate in collaboration with professor of zoology at the University of Toronto, Henry Regier, and fishery scientist Richard Ryder. The walleye (*Sander vitreus*), another valued fish species in Lake Erie, had in recent years seen fluctuating population sizes. Reflecting on a high point in walleye numbers in the early 1950s, they stated that this “[…] level of abundance seems to have been the consequence of a unique set of factors that are not likely to occur simultaneously again. In fact, we see in such abundance not a symptom of health of the whole fish system but rather a system disruption. A valid analogue might be a sharp increase in a man’s weight leading to obesity not being indicative of health but rather of an undiagnosed disease.”^58^ At the time, this “undiagnosed disease” causing the sharp fluctuations in walleye populations was identified primarily as unregulated fisheries and secondarily pollution and the introduction of invasive species such as smelt (*Osmerus mordax*) and alewife (*Alosa pseudoharengus*), which together caused cascading effects within the fish populations, leading to the walleye’s decline. In its concluding section to the summary, the authors

…suggest that basic management policy be geared to stabilizing the fish **system**. The present policy of fishing heavily whatever species is currently abundant appears to increase the amplitude of those fluctuations that do occur, creating unstable and often economically inefficient conditions in the industry. The sudden occurrence of a very large year class of a valued species should be taken as a symptom of serious trouble in the ecosystem and not the source of glee that it appears to be under the current approach. […] Our analysis is intended as a first step toward a more balanced, more rational, and we hope more successful systems approach to management of the Lake Erie fishery.^59^

During the same period, the sea lamprey invasion also became described in ecosystemic terms. This new problematization of the sea lamprey as an invasion with detrimental effects on a complex and traditionally healthy ecosystem was a response to the rethinking of the Great Lakes as a threatened environment. In the committee minutes and publications commissioned by the GLFC, the ecosystem concept started to occasionally appear during the 1960s in discussions related to the sea lamprey, however it was not often explained. Instead, its definition was presumed to be known by the audience. During these years, the ecosystem concept became popularized and debated within the environmentalist movement and the American scientific community. In “Rehabilitating Great Lakes Ecosystems,” an influential report published in 1979 that informed subsequent Great Lakes management strategies and written by various prominent American and Canadian fishery biologists, the term ecosystem was defined as

an essentially natural complex of interlinking entities and processes which operate within some part of physical space. Physical space is simply a context which can be bounded by reference to geographic, hydrologic and atmospheric factors. Boundaries around a particular ecosystem usually cannot be specified precisely, but this is not crucial. In the case of the Great Lakes for example, the whole drainage basin of all the lakes can be viewed as an ecosystem for some purposes. For other purposes, it might be more appropriate to choose a “sub-system” such as one of the lakes, or a bay […], or a small lagoon on the point itself. The real focus of interest in ecosystems is on the self-organizing, dynamic processes of the system rather than on its boundaries.^60^

Although ambiguities remained, Great Lakes fishery biologists seemed to refer to an ecosystem as a closed system in which organisms of a community are part of a larger physical system in which they live, and from which they could not be separated.^61^ External (human caused) disturbances could however upset the systemic balance, potentially with far reaching consequences to public health and wildlife. Such a systemic disbalance became understood as an unhealthy situation.

Several aquatic crises played a major role in shaping public and scientific perception of the Great Lakes as a heavily disturbed ecosystem. Firstly, in the wake of Rachel Carson’s *Silent Spring*, published in 1962, the effects of the agricultural pesticide DDT was regarded as a major factor of pollution in the Great Lakes, introduced via agricultural activities near the lake shores and tributaries, causing the accumulation of toxins in fish.^62^ A second pollution issue came to the fore in 1970, when the Canadian Federal Department of Fish and Forestry temporarily banned the sale of Great Lakes freshwater fish due to high amounts of mercury measured.^63^ This led to a major controversy involving the Dow Chemical Company, a prominent industrial producer with a factory near Detroit, regarded to have caused the crisis.^64^ These issues highlighted that anthropogenic factors could disrupt Great Lakes ecosystems, threatening both public health and health of wildlife. With the signing of the Great Lakes Water Quality Agreement between the US and Canada in 1972, water quality became a focus point for scientific interest and environmental protection in the Great Lakes.^65^

During the same period, heaps of dead alewives (*Alosa pseudoharengus*) – another invasive fish species utilizing the same pathway as the sea lamprey – were noticed on lake shores and in the harbor of Detroit.^66^ The unpleasant sight and smell of these “die-offs” became a topic of discussion, specifically the question what had caused these sudden episodes.^67^ Later research would point at various causes, a prominent one being unfavorable temperature fluctuations.^68^ Also, the introduction of millions of lake trout yearlings via local hatcheries did not lead to the direct establishment of a large lake trout population. Since the sea lamprey population was significantly reduced, what caused the difficulty in re-establishing the lake trout population?

In terms of sea lamprey research, the 1970s were very much a period of transition from traditional Great Lakes fishery science based on fish population and community studies to understanding fisheries as part of a more complex environment. It was argued that fish population shifts should be understood as the consequence of multiple environmental and inter-specific causes.^69^ One illustrative example of this is the work by Stanford H. Smith. In 1972 he published multiple articles as part of the proceedings of the Salmonid Communities in Oligotrophic Lakes (SCOL) symposium. This symposium, held at Geneva Park, Ontario in July 1971, was convened by Henry Regier, a University of Toronto fishery biologist who previously contributed to the walleye study and was driven by strong ecological concerns.^70^ The symposium aimed to expand a “population dynamics approach” to fisheries by considering “other stresses besides exploitation.” This included not only the introduction of exotic species, but also the effects of various forms of environmental pollution, including “enrichment by plant nutrients” (fertilizers) and industrial pollution, such as the dumping of sawmill dust. Furthermore, shore construction and water engineering activities such as the construction of dams, the drainage of swamps and marshes, and deforestation were considered as detrimental factors. Together, they led to compounding effects that caused the reduction of water flows and increased water temperatures. This resulted in reductions of various fish species, especially in Great Lakes bays, marshes, and tributaries.^71^ At the same time, Smith was quoted in a newspaper article titled “Don’t Just Blame the Alewives” that these human-caused habitat alterations were even favorable for the alewives and sea lampreys: “If it were not for the temperature change that occurred in tributaries in the Lake Ontario basin,” he argued, “we would not have one alewife or one sea lamprey anywhere in the Great Lakes.”^72^ Smith’s work is illustrative of attempts to broaden the epistemological scope of fishery research and encourage an engagement with complex ecosystemic interactions.

**Figure 2.**
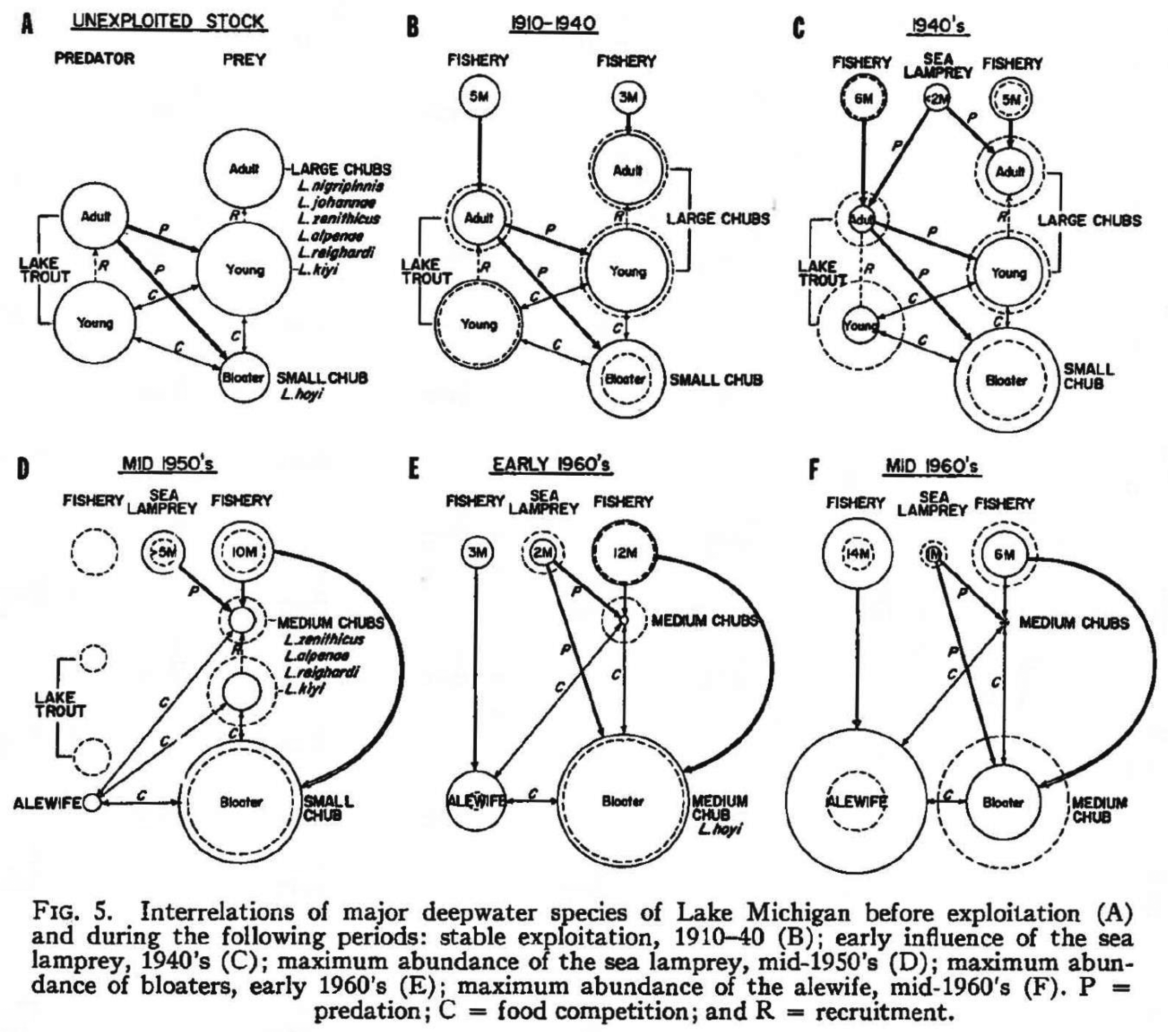
Smith, “Species Succession and Fishery Exploitation in the Great Lakes,” 682. Diagrams that illustrate the changing relations between Great Lakes fish species in Lake Michigan before and after intensive fisheries and sea lamprey introduction. Recruitment refers to young fish transitioning to an older, larger life stage.

In his 1968 article “Species Succession and Fishery Exploitation in the Great Lakes,” Smith had already discussed how the composition of Great Lakes fish populations had been severely disrupted since the late nineteenth century.^73^ He utilized the single case of Lake Michigan fish populations to show that a natural balance in predator-prey relationships between lake chubs, (*Coueius plumbeus*) a group of smaller deep-water fish, and larger lake trout were upset by compounding effects of overfishing and sea lamprey predation (see Figure 2). Smith argued that during the 1950s, lake trout populations had plummeted and both fisheries and sea lamprey shifted their attention to other fish and prey. Sea lamprey not only shifted to lake whitefish, white suckers, and walleyes, but also larger chubs. Chubs subsequently bore the brunt of both fishing and sea lamprey predation. Smith concluded that these factors led to a rapid process of succession, a change in population composition, including size and growth rate.

This was thus caused by environmental changes, (over)fishing, and sea lamprey predation, although he argued that it was difficult to differentiate among the three.^74^ Smith continued that these events combined “produced a great instability in the balance of deepwater [sic] species, created a strong pressure against the large chubs, and favored bloaters […].”^75^ Bloaters (*Coregonus hoyi*) were traditionally the smallest and slowest growing chub and by 1960-1961 constituted ninety-three to- ninety-five percent of chubs taken. Smith concluded by warning that the recent biological changes of the bloater stocks may have been indicators of a pending population collapse. He argued that the restoration of the Great Lakes ecosystem required “careful regulation of the kinds and numbers of predators, and the reestablishment of a multi-species complex of prey species.”^76^

He followed up on this last point in 1972. In “The Future of Salmonid Communities in the Laurentian Great Lakes,” part of the SCOL proceedings, he reiterated that the ability of the Great Lakes sea lampreys and alewives to greatly reduce their food supply and thereby contribute to their own population reduction was illustrative of ecosystem instability.^77^ Subsequently he explained different scenarios for Great Lakes ecosystem restoration. Firstly, no management action might lead to a new equilibrium. This could either result in 1) the establishment of a new stable ecosystem in which the sea lampreys and alewives could find their place and their populations would be dampened, or 2) the disappearance of the sea lampreys and alewives, or 3) the disappearance of native fish, “eventually leading to an entirely new fishery ecosystem.”^78^ Alternatively, the outcome of a management program including sea lamprey control and salmonid predator stocking (including lake trout) was highly uncertain according to Smith, due to the uncertainty of whether salmonid stocking would lead to an upsurge in sea lamprey numbers, whether the stocked fish could be maintained, and whether other reduced fish populations, including lake whitefish, lake herring, deepwater ciscoes, and emerald shiners (*Notropis atherinoides*) were able to recover.^79^ Lastly, Smith emphasized that “unfavorable water quality […] pose[s] the most serious and limiting threat to the success of any program to improve or restore the fishery resources of the Great Lakes.”^80^ This would require a carefully coordinated effort of various managerial interventions.

Although Smith based his publication on traditional fishery data and population ecology, he discussed the sea lamprey as one of a multivariate of different factors producing an unhealthy fishery ecosystem that he understood to be on the verge of collapse. He presented the sea lamprey as an invasive species that both upset the fishery ecosystem while also being affected by the Great Lakes environmental conditions. However, an important epistemological tension remained: the sea lamprey was both defined as an external factor that upsets the valued fish populations, while itself also becoming part of the Great Lakes ecosystem.

## Integrated Pest Management (IPM): A New Systemic Approach to Sea Lamprey Control

During the 1960s, GLFC scientists continued to discuss ongoing sea lamprey research and control operations in various lake committees. These committees took shape as advisory bodies for the Great Lakes Fishery Commission board, and each of the Great Lakes received a separate committee.^81^ According to Marc Gaden, who published extensively on the history of the GLFC and Great Lakes cross-border policy, “the meetings were useful as a way to share information but beyond that they inspired little strategic action,” although their main contribution was “to create an ongoing process of interactions among like-minded professionals and to begin the development of a culture of cooperation in the region.”^82^ A separate Scientific Advisory Committee was established with the foundation of the GLFC to provide scientific support for fishery management policies..

While reading the minutes of this Scientific Advisory Committee, one can notice how during the late 1960s and 1970s for the first time the sea lamprey was mentioned in broader conversations on the future role of the GLFC in Great Lakes water management issues. The documents in the appendix to the Scientific Advisory Committee meeting that was held in Ottawa on 18 June 1973 are an example of this. The central topic of the meeting was the need for drafting a new research prospectus that “should provide for broad economic and sociological, as well as biological and environmental aspects of resource development and management.”^83^ Of the annexes, a position paper, titled “The Great Lakes Fishery Commission – Structures and Functions,” drafted on 8 June 1973 stated that,

The GLFC was structured to address one major problem of 1955 – the lamprey. Secondarily it was granted a research co-ordinating role; it has also gradually developed an initiatory function with research. Are the present structure and functions adequate for 1973’s problems? […] If not […] then how might the GLFC be restructured? Or should it perhaps be phased out of existence to permit the development of a new institution?^84^

Another document, drafted on 30 May 1973 stated that,

the characteristics of the Great Lakes fish populations have changed greatly since the Convention on Great Lakes Fisheries was signed […]. New management approaches and philosophies have evolved reflecting advances in fishery science […]. Also, the fisheries have become a less discrete resource and are inseparably entwined with the full range of uses of the Great Lakes for recreation, navigation, domestic and industrial water supply and waste disposal […].^85^

Although parts of these quotes might seem ambiguous, they express a sense of urgency in adjusting management directions to the complexity of the new environmental challenges.

Simultaneously, a new generation of fishery managers that had been informed by novel directions in ecology in the 1960s started to push for the inquiry of new approaches to sea lamprey control. On 27 November 1970, Lee H. Hanson, at the time a Hammond Bay Biological Station scientist, provided an ambitious discussion paper on integrated pest management (IPM) as a new direction for dealing with the sea lamprey. He introduced this as “a pest population management system that utilizes all suitable techniques in a compatible manner to eliminate or reduce pest populations and maintain them at levels below those causing economic injury. Integrated control achieves this ideal by harmonizing techniques in an organized way, by making the control practices compatible, and by blending them into a multifaceted, flexible, evolving system.”^86^ He continued with outlining various promising control techniques that might be considered in the near future and differentiated between biological control and genetic control. Hanson summarized biological control as “an indefinable label applied to fighting biology with biology,” including the introduction of pathogens or other animals that would have a detrimental effect on the sea lamprey.^87^ The GLFC could explore the introduction of a predator from the American east coast to eat the sea lamprey or alternatively introduce another, non-parasitic lamprey species that did not cause any damage to the valued Great Lakes fish and would compete with the sea lamprey for the same food source. Furthermore, Hanson stressed the need for extensive ecological studies before and after the introduction of biological control species to monitor the process. For genetic control, the development of a “sterile male program” was suggested – which would mean the catch, sterilization, and subsequent release of upstream migration male sea lampreys before spawning, leading to ineffective reproduction. Lastly, Hanson also considered the introduction of a chemical sterilant in spawning streams.^88^ He envisioned these strategies to complement TFM, since although TFM would lead to low sea lamprey population numbers it would not allow for the full eradication of the sea lamprey in the Great Lakes.

Hanson and his colleagues followed the new generation of fishery scientists, and the sea lamprey invasion became newly perceived as one of the various causes of a disbalanced and ailing ecosystem. Hanson implied this in his paper on IPM, by stating that,

It is generally conceded that the best subjects for biological control are organisms accidently introduced into a new region without the large complex of parasites and predators that attack them in their native homes [the sea lamprey is implied here]. These organisms may be slow to attract parasites and predators in the new region; they often find large and virtually unexploited source of food, […]. Compared with the complex interrelations existing between an animal and its environment when the two have evolved together for countless generations these immigrant animals present a relatively simple problem; their relations with their environment are incompletely established and are amenable to manipulate.^89^

With this contextualization, the sea lamprey was represented as part of an ailing ecosystem, although one that should be removed and only allowed to exist in its native environment.

By the time of the publication of Hanson’s discussion paper, IPM had already become a standardized format in pest control. Originally developed by applied entomologists at Californian universities, IPM was envisioned as a response to the need for new approaches in pest eradication due to certain insects becoming resistant to pesticides and the DDT scandal that had led to policies restricting or banning the use of DDT.^90^ The GLFC scientists regarded IPM as a favorable alternative by utilizing various control strategies, preferring biological control over chemical control (only utilizing toxicants as a last resort), that were adjusted to the local ecological requirements.^91^ It was presented as the most recent trend in scientific pest control, mitigating any damaging side effects to public health or the natural environment, resulting in successful pest eradication. For the GLFC staff, it promised a more effective control strategy that could draw on their scientific expertise of Great Lakes fish populations. While it connected to the previous experimental and pragmatic approach to sea lamprey control of the late 1940s and 1950s, in which various forms of mechanical control and chemical control were explored and combined to organize effective control, it also allowed for a new discussion on how to broaden sea lamprey control strategies and reevaluate the use of TFM therein.

The latter was certainly necessary since TFM itself had become scrutinized for potential environmental side effects. Various alarming reports had been made on local fish deaths after TFM application, and the American Environmental Protection Agency (EPA) requested in the early 1970s an elaborate study before the chemical could be re-registered as a pesticide. During this time of uncertainty on the health risks of pesticides, IPM provided a timely alternative strategy for continuous sea lamprey control.^92^

An IPM approach to sea lamprey control remained an important topic of discussion during the 1970s and became the dominant scientific and managerial direction driving sea lamprey research and control by 1980. In the major report “Rehabilitating Great Lakes Ecosystems,” followed by the “Joint Strategic Plan for Management of Great Lakes Fisheries” (commonly named The Plan) in June 1981, the sea lamprey invasion was represented as one of the major threats to Great Lakes ecosystem services.^93^ The GLFC “recognizes that any impact on a part of the system may to some degree affect an entire lake, connecting channels, and even the entire basin.” In another section, it stated that “stresses affecting fishery resources rarely act singly, often have complex interactions and often impact several levels of the aquatic ecosystem so that remedial management must address problems on a comprehensive whole-system basis.”^94^ In “Rehabilitating Great Lakes Ecosystems,” the notion of health was used both in relation to the threat of toxins to public health and a healthy Great Lakes ecosystem as a whole: “[…]perhaps the greatest values of ecosystem rehabilitation derive from the fact that people strongly value ‘healthy’ productive Great Lakes ecosystems. They feel better off, perhaps more secure in the knowledge that healthy Great Lakes exist, and will be available in case they or their offspring wish to use them.” This quote illustrates how a traditional fishery resource discourse had become connected to ecosystemic health. At the same time, in sections concerning the sea lamprey invasion, the traditional resource perspective on fisheries remained dominant: “The fishery resources have been diminished and much altered through exploitation by man, degradation of habitat and the introduction or invasion of exotic biota” and “the parasitic sea lamprey, although significantly controlled in most areas, continues to have an adverse effect on high value species.”^95^ These services included commercial use for fisheries, recreational use, and land use affecting the Great Lakes.

The work of Great Lakes fishery scientists who framed the sea lamprey invasion as an ecosystemic issue became the basis for the period of the most extensive research activities on sea lamprey to date that started in the early 1980s. During a series of International Sea Lamprey Conferences (SLIS), firstly organized in 1978 and roughly organized every ten years, an international community of sea lamprey scientists came together to discuss new avenues in sea lamprey ecology and research for control purposes.^96^ IPM had become the preferred management direction, and should be supported by “systems analysis” aided by quantitative models that would predict the outcome of various combined management interventions.^97^

Genetic control became a prominent area of research – firstly via the sterile male program developed during the 1970s and 1980s by Hanson and others. Another new research avenue became the topic of sea lamprey pheromones, and how they could be used as attractants and repellants for control purposes.^98^

## Conclusion

After its arrival in the Great Lakes, the sea lamprey was initially an unknown fish, but after the establishment of a stable population in Lake Huron and Lake Michigan it became regarded as the single most pressing Great Lakes fishery issue and it was accused of causing the plummeting lake trout catch numbers in the early 1940s. Initially, the sea lampreys were represented by a coalition of Great Lakes fishermen, fishery biologists, politicians, and journalists as an alien threat, consisting of aggressive fish that quickly expanded their range, produced high numbers of offspring, and ferociously attacked defenseless and healthy food fish, in particular the lake trout. In times of an already ailing fishing industry, the sea lamprey introduction became successfully singled out, while complex ongoing discussions on overfishing and pollution were moved to the background.

A coalition of Great Lakes fishery biologists pushed the case in various US Senate committee hearings to acquire continuous funding for sea lamprey research and control activities. Not only did these discussions lead to the signing of the Convention on Great Lakes Fisheries and the foundation of the GLFC, they also reformulated the sea lamprey problem as a national food security issue that warranted federal support. This marked the start of elaborate sea lamprey life history studies, leading the Great Lakes fishery scientists to understand the sea lamprey in terms of the characteristics that made it invasive: its migratory, predatory and reproductive behavior. What followed was a period of arduous mechanical control, utilizing weirs and electric fences.

The introduction of TFM, an effective lampricide that targeted the sea lamprey ammocetes in 1958, marked the beginning of a reconsideration of what constituted the sea lamprey issue. The toxin was effective in reducing the sea lamprey population by ninety percent, leading to the prospect of Great Lakes fishery rehabilitation. This meant that the issue was transformed into an issue of maintaining low population numbers, instead of eradicating adult sea lampreys. In the 1960s a discussion emerged on the necessity of continuous TFM application and its potential effects on healthy native fish and public health. Now it became regarded as a potential risk, with the prospect of a sudden sea lamprey upsurge.

During the second half of the 1960s and in the wake of the DDT controversy, the Great Lakes received interest from a new generation of environmentalists and scientists who wanted to reduce Great Lakes pollution. Fishery management was affected by this due to the Great Lakes being reinterpreted as an unhealthy ecosystem. As Stanford H. Smith’s work exemplifies, traditional fishery research based on fish catch data and complex fish population studies became increasingly inspired by a discussion on the Great Lake ecosystem. The exact characteristics and extent of this Great Lakes ecosystem was a topic of discussion, in a period in which the concept itself was negotiated by ecologists. In fact, it was utilized as a catch-all phrase that incorporated many different biotic and abiotic interactions. The Great Lakes fish populations now became regarded as part of a complex multivariate system, in which the fish could be affected by various cascading effects. Improving the health of the Great Lakes meant not only understanding this complex ecosystem, but also introducing managerial interventions that went beyond traditional fishery regulations and sea lamprey control in service of restoring an ecological balance. IPM proved to be suitable for this purpose, and allowed for the incorporation of ongoing chemical control while making Great Lakes Fishery Committee scientists cognizant of the need for further research to come up with additional control strategies.

Although the ways in which the sea lamprey was problematized changed, the sea lamprey itself continued to be represented as a problematic outsider. In addition to a traditional perception of the sea lamprey in terms of population ecology (life history, migration, and interactions with other fish species) the sea lamprey was by the late 1960s seen as an introduced species that affected other Great Lakes fish as part of a complex environment. The system was also affected by humans who caused environmental disruptions such as overfishing, pollution, and other accidental species introductions. The sea lamprey became thus understood as part of an ailing ecosystem, while it continued to be framed as an alien invader that had to be removed to restore Great Lakes ecosystem health. Indeed, the ecosystem discussion did not cause an epistemological shift in how the sea lamprey itself was perceived. It continued to be regarded as an outsider that never ought to be in the Great Lakes, and that had never became valued life as a new resident. This stands in stark contrast to other fish species introduced in the 1960s and 1970s that were seen as valuable additions to the Great Lakes fish population.

The new problematization of the sea lamprey as an ecosystemic health issue allowed the continuation and expansion of past scientific and managerial practices. No paradigmatic shift occurred that could have resulted in reconsidering the sea lamprey an inhabitant of the Great Lakes. Instead, the case shows how influential the initial conceptualization of the sea lamprey as a threat to Great Lakes fisheries was and resonated in later managerial discussions. The renewed problematization of pest or invasive animals in terms of ecosystem health first and foremost allowed for the continuation of past directions in environmental management, in tandem with the exploration of new additional research avenues.

The sea lamprey can be considered an important test case for fishery scientists and managers during the 1930s and 1970s, while nowadays it can be regarded as an illustrative case for animal invasion history. At the time, it allowed scientists to study the sea lamprey in support of an elaborate eradication program, and come up with various control strategies, during a period in which not only fishery science considerably changed but also invasion ecology emerged. For the historian nowadays it is a case that helps illustrate how animal invasions were no longer predominantly perceived as alien threats to human valued activities such as agriculture or fisheries, but also how incrementally various notions of health (food security, health of individual and ecosystem health) became connected to animal invasions.

## Footnotes

1 Cory O. Brant, *Great Lakes Sea Lamprey: The 70 Year War on a Biological Invader* (Ann Arbor: University of Michigan Press, 2019), 40; Martin V. Melosi, *Water in North American Environmental History* (London: Routledge, 2022), 109-119.

2 Brant, *Great Lakes Sea Lamprey*, 40.

3 Ibid., 33-36.

4 Ibid., 12-13.

5 Ibid., 96-97.

6 Marc Gaden, Cory O. Brant, and Robert Lambe, “The History behind the Canada-U.S. Convention on Great Lakes Fisheries: A Seven-Decade Effort,” *Laurentian: Journal of the Great Lakes Fishery Commission* 1 (2022): 1-97.

7 Charles Elton, *The Ecology of Invasions by Animals and Plants*, with contributions by Daniel Simberloff and Anthony Ricciardi (Cham: Springer Nature, 2020 [1958]).

8 Stephen Bocking, *Ecologists and Environmental Politics: A History of Contemporary Ecology* (Morgantown: West Virginia University Press, 2017); Stephen Bocking, “Fishing the Inland Seas: Great Lakes Research, Fisheries Management and Environmental Policy in Ontario,” *Environmental History* 2 (1997): 52-73; Margaret B. Bogue, *Fishing the Great Lakes: An Environmental History 1783-1933* (Madison: University of Wisconsin Press, 2000); Brant, *Great Lakes Sea Lamprey*; John C. Hudson and Susy S. Ziegler, “Environment, Culture and the Great Lakes Fisheries,” *Geographical Review* 104 (2014): 391-413; Marc Gaden, “Bridging Jurisdictional Divides: Collective Action Through a Joint Strategic Plan for Management of Great Lakes Fisheries” (PhD diss., University of Michigan, 2007); Gaden, Brant, and Lambe, “The History behind the Canada-U.S. Convention on Great Lakes Fisheries”; Lynne Heasley and Daniel Macfarlane, ed., *Border Flows: A Century of the Canadian-American Water Relationship* (Calgary: University of Calgary Press, 2016); Nancy Langston, *Sustaining Lake Superior*: *An Extraordinary Lake in a Changing World* (New Haven: Yale University Press, 2017); Jill Wingfield, Cory Brandt, Randy Eshenroder, Marc Gaden, Andrea Miehls, Michael Siefkes, “100 Years of Sea Lampreys Above Niagara Falls: A Reflection on What Happened and What We Learned,” *Journal of Great Lakes Research* 47 (2021): 1844-1848.

9 John Hannigan, *Environmental Sociology* (Abingdon: Routledge, 2023).

10 Ibid., 53.

11 Angela Cassidy, *Vermin, Victims and Disease*: *British Debates over Bovine Tuberculosis and Badgers* (Cham: Palgrave MacMillan, 2019), 3; Sarah Jansen, *Schädlinge. Geschichte eines wissenschaftlichen und politischen Konstrukts, 1840-1920* (Frankfurt: Campus Verlag, 2003); Jules Skotnes-Brown, *Segregated Species: Pests, Knowledge and Boundaries in South Africa, 1910-1948* (Baltimore: Johns Hopkins University Press, 2024).

12 Christos Lynteris, ed., *Framing Animals as Epidemic Villains: Histories of Non-Human Disease Vectors* (Cham: Palgrave Macmillan, 2019).

13 Langston, *Sustaining Lake Superior*, 170.

14 Peter Coates, *American Perceptions of Immigrant and Invasive Species: Strangers on the Land* (Berkeley: University of California Press, 2006); Matthew Holmes, “A Plague of Weasels and Ticks: Animal Introduction, Ecological Disaster, and the Balance of Nature in Jamaica, 1870-1900,” *British Journal for the History of Science* 56 (2023): 391-407.

15 Raf De Bont, “Hamster Numbers: Biopolitics and Animal Agency in the Dutch fields, circa 1870-present,” *History and Philosophy of the Life Sciences* 43 (2021): 1-25; Peter Coates, “The Muskrat’s New Frontier: The Rise and Fall of an American Animal Empire in Britain,” *Environmental History* 25 (2020): 207-236; Jodi Frawley and Iain McCalman, ed., *Rethinking Invasion Ecologies from the Environmental Humanities* (London: Routledge, 2014); Ashanti Ke Ming Shih, “Invasive Ecologies: Science and Settler Colonialism in Twentieth-Century Hawai’i” (PhD diss., University of Chicago, 2020); Jeannie N. Shinozuka, *Biotic Borders: Transpacific Plant and Insect Migration and the Rise of Anti-Asian Racism in America, 1890-1950* (Chicago: University of Chicago Press, 2022).

16 Matthew D. Turner, “’Forbidden Fish’: Did King Henry I Die of Lamprey Poisoning?” *Cureus* 15: 5 (2023): 1-5.

17 David A. Close, Martin S. Fitzpatrick, and Hiram W. Li, “The Ecological and Cultural Importance of a Species at Risk of Extinction, Pacific Lamprey,” *Fisheries Magazine* 27: 7 (2011): 19-25.

18 Simon Henry Gage, *The Lake and Brook Lampreys of New York. Especially those of Cayuga and Seneca Lakes* (Ithaca, NY, 1893); Simon Henry Gage, “The Lampreys of New York State - Life History and Economics,” *Biological Survey of the Oswego River System, state of New York Conservation Department, Supplement to the 17*^*th*^  *Anniversary Report* (1928): 158-191; Stanford H. Smith, “Early Changes in the Fish Community of Lake Ontario,” *Technical Report* 60 (April 1995): 19-25; H.A. Surface, “The Lampreys of Central New York,” *Bulletin of the United States Fish Commission* 17 (1897): 209-215.

19 Surface, “Lampreys of Central New York,” 213.

20 “Sea Lampreys Spread to All Great Lakes. Spawning Run of Parasites Found In Ocqueoc River in Michigan,” *Great Lakes Journal*, August 1937.

21 “Dread Sea Lampreys in First Spawn Run,” *Milwaukee Journal*, 15 August 1937.

22 Ibid.

23 Brant, *Great Lakes Sea Lamprey*, 103.

24 Bogue, *Fishing the Great Lakes*.

25 Carl L. Hubbs and T.E.B. Pope, “The Spread of the Sea Lamprey through the Great Lakes,” *Transactions of the American Fishery Society* 66 (1937): 172.

26 John Van Oosten, “Some Fisheries Problems on the Great Lakes,” *Transactions of the American Fisheries Society* 59 (1929): 63-85.

27 Bogue, *Fishing the Great Lakes*.

28 Gaden, Brant, and Lambe, “The History behind the Canada-U.S. Convention on Great Lakes Fisheries,” 2-3.

29 Brant, *Great Lakes Sea Lamprey*, 65.

30 John Van Oosten, “The Disappearance of the Lake Erie Cisco – A Preliminary Report,” *Transactions of the American Fisheries Society* 60 (1930): 204-214.

31 Brant, *Great Lakes Sea Lamprey*, 60-70.

32 Hubert R. Gallagher, A.G. Huntsman, D.J. Taylor, and J. Van Oosten, ed., *Report of the International Board of Inquiry for the Great Lakes Fisheries* (Washington, DC, 1943).

33 Brant, *Great Lakes Sea Lamprey*, 77; US Congress, House of Representatives, Committee on Merchant Marine and Fisheries, *Menace of the Sea Lamprey*, 79^th^ Congress, 2^nd^ session, 1946, 4-9.

34 Andrew Muir, Michael J. Hansen, and Charles C. Krueger, “Re-establishing Lake Trout in the Laurentian Great Lakes: Past, Present and Future,” in *Great Lakes Fishery Policy and Management: A Binational Perspective,* ed. William W. Taylor, Abigail J. Lynch, and Nancy J. Leonard (East Lansing: Michigan State University Press, 2013), 536-541.

35 US Congress, House of Representatives, *Menace of the Sea Lamprey*, 1-15.

36 Ibid., 2.

37 Ibid., 8.

38 Ibid., 18.

39 Elinor Forsythe, “Condensed Transcript of Proceedings of the Great Lakes Sea Lamprey Conference, November 14 and 15 1946, Ann Arbor, Michigan,” Great Lakes Fishery Commission Archives, 1-33.

40 John Van Oosten, “The Sea Lamprey – A Threat to the Great Lakes Fisheries,” 7 October 1949, 1-6, Great Lakes Fishery Committee Archives.

41 US Congress, House of Representatives, Committee on Merchant Marine and Fisheries, Subcommittee on the Fisheries and Wildlife Conservation, *Commercial Fishing in the Great Lakes Area*, 81^st^ Congress, 1^st^ session, 1949, 64.

42 Ibid., 83.

43 Stanford H. Smith, “Species Succession and Fishery Exploitation in the Great Lakes,” *Journal of the Fisheries Research Board of Canada* 25 (1968): 667-693.

44 Langston, *Sustaining Lake Superior*, 169-171.

45 US Congress, House of Representatives, *Commercial Fishing in the Great Lakes Area*, 73.

46 Ibid., 12.

47 Vernon Applegate, *Natural History of the Sea Lamprey, Petromyzon marinus, in Michigan* (Ann Arbor: Michigan University Press, 1950).

48 Brant, *Great Lakes Sea Lamprey*, 12-13.

49 Gaden, Brant, and Lambe, “The History behind the Canada-U.S. Convention on Great Lakes Fisheries.”

50 Frederick Rowe Davis, *Banned: A History of Pesticides and the Science of Toxicology.* (New Haven: Yale University Press, 2014).

51 Brant, *Great Lakes Sea Lamprey*, 120.

52 Robert E. Lennon, “Feeding Mechanism of the Sea Lamprey and its Effects on Host Fishes,” *Fish and Wildlife Service Fishery Bulletin* 98 (1954): 284-286; Philip S. Parker and Robert E. Lennon, “Biology of the Sea Lamprey in its Parasitic Phase,” *Fish and Wildlife Service Research Report* 44 (1956): 3.

53 A.L. Prichard, “Report of the Chairman,” Great Lakes Fishery Commission Annual Meeting, 17-18 June 1964: 1-4, Great Lakes Fishery Commission Archives.

54 Brant, *Great Lakes Sea Lamprey*, 133

55 Bernard R. Smith, J. James Tibbles, and B.G.H. Johnson, “Control of the Sea Lamprey (*Petromyzon Marinus*) in Lake Superior, 1953-70,” *Great Lakes Fishery Commission Technical Report* 26 (March 1974): 35-36.

56 “Report of Scientific Advisory Committee,” Great Lakes Fishery Commission, Annual Meeting, 22 June 1965, 3, Great Lakes Fishery Commission Archives; Lee Emery, “Review of Fish Species Introduced into the Great Lakes, 1819-1974,” *Great Lakes Fishery Commission Technical Report* 45 (April 1985): 6-11.

57 Kristin M. Szylvian, “Transforming Lake Michigan into the ‘World’s Greatest Fishing Hole’: The Environmental Politics of Michigan’s Great Lakes Sport Fishing, 1965-1985,” *Environmental History* 9 (2004): 102-127.

58 Henry A. Regier, Vernon C. Applegate, Richard A. Ryder, “The Ecology and Management of the Walleye in Western Lake Erie,” *Great Lakes Fishery Commission Technical Report* 15 (May 1969): 2.

59 Ibid., 4, emphasis in the original.

60 George R. Francis, John J. Magnuson, Henry A. Regier, and Daniel R. Talhelm, “Rehabilitating Great Lakes Ecosystems,” *Great Lakes Fishery Commission Technical Report* 37 (December 1979): 12.

61 Sharon E. Kingsland, *The Evolution of American Ecology, 1890-2000* (Baltimore: Johns Hopkins University Press, 2005), 184.

62 Langston, *Sustaining Lake Superior*.

63 Simone Müller, “Corporate Behaviour and Ecological Disaster: Dow Chemical and the Great Lakes Mercury Crisis, 1970-1972,” *Business History* 60 (2017): 407.

64 Ibid., 400.

65 Langston, *Sustaining Lake Superior*, 186-196.

66 Szylvian, “Transforming Lake Michigan,” 112.

67 “The Alewife Explosion: The 1967 Die-Off in Lake Michigan. A Report by the Federal Water Pollution Control Administration, Great Lakes Region,” 25 July 1967.

68 Robert O’Gorman and Thomas J. Stewart, “Ascent, Dominance and Decline of the Alewife in the Great Lakes: Food Web Interactions and Management Strategies,” in *Great Lakes Fisheries Policy and Management: A Binational Perspective*, ed. W.W. Taylor and C.P. Ferreri (East Lansing, MI: Michigan State University Press, 1999), 494.

69 Bocking, “Fishing the Inland Seas,” 62; Bocking, *Ecologists and Environmental Politics*, 167-178.

70 Bocking, ”Fishing the Inland Seas,” 61; Stanford H. Smith, “The Future of Salmonid Communities in the Laurentian Great Lakes,” *Journal of the Fisheries Board of Canada* 29 (1972): 951-957; Stanford H. Smith, “Factors of Ecologic Succession in Oligotrophic Fish Communities of the Laurentian Great Lakes,” *Journal of the Fisheries Board of Canada* 29 (1972): 717-730.

71 Kenneth H. Loftus and Henry A. Regier, “Introduction to the Proceedings of the 1971 Symposium on Salmonid Communities in Oligothrophic Lakes,” *Journal of the Fisheries Board of Canada* 29 (1972): 613-616.

72 Miles McNamara, “Don’t Just Blame Alewives,” *Milwaukee Journal*, 12 March 1972.

73 Smith, “Species Succession and Fishery Exploitation in the Great Lakes.”

74 Ibid., 681.

75 Ibid., 684.

76 Ibid., 690.

77 Smith, “The Future of Salmonid Communities,” 953.

78 Ibid.

79 Ibid., 954.

80 Ibid., 956-957.

81 Gaden, “Bridging Jurisdictional Divides,” 42-45.

82 Ibid., 45.

83 “Meeting of the Scientific Advisory Committee, Report and Recommendations,” 18 June 1973, Great Lakes Fishery Commission Archives.

84 H. Regier, “The Great Lakes Fishery Commission - Structures and Functions,” Great Lakes Fishery Commission, Scientific Advisory Committee, 18 June 1973,1-3, Great Lakes Fishery Commission Archives.

85 “Recommendation of the Scientific Advisory Committee. Review of Great Lakes Fishery Convention and Commission,” Meeting of the Scientific Advisory Committee, 18 June 1973, 1-3, Great Lakes Fishery Commission Archives.

86 Lee H. Hanson, “Prospects for the Integrated Control of the Sea Lamprey,” Meeting of the Scientific Advisory Committee, 22 June 1971, 1-23, Great Lakes Fishery Commission Archives, emphasis in the original.

87 Ibid., 1.

88 Ibid., 11-20.

89 Ibid., 2-3.

90 Richard C. Sawyer, *To Make a Spotless Orange: Biological Control in California* (Ames: Iowa State University Press, 1996), 258.

91 John H. Perkins, *Insects, Experts, and the Insecticide Crisis: The Quest for New Pest Management Strategies* (New York: Plenum Press, 1982); Terrance D. Hubert, James Miller, and Dale Burkett, “A Brief Introduction to Integrated Pest Management for Aquatic Systems,” *North American Journal of Fisheries Management* 41 (2019): 264-275.

92 “Great Lakes Fishery Commission, Annual Report for the year 1970,” 1972.

93 Francis et al., “Rehabilitating Great Lakes Ecosystems”; “A Joint Strategic Plan for Management of Great Lakes Fisheries,” June 1981.

94 “A Joint Strategic Plan,” 1-4.

95 Ibid., 3.

96 Bernard R. Smith, “Introduction to the Proceedings of the 1979 Sea Lamprey International Symposium (SLIS),” *Canadian Journal of Fisheries and Aquatic Sciences* 37 (1980): 1585-1587.

97 Patrick F. Lett, F.W.H. Beamish, and G.J. Farmer, “System Simulation of the Predatory Activities of Sea Lampreys (*Petromyzon marinus*) on Lake Trout (*Salvelinus namaycush*),” *Journal of the Fisheries Board of Canada* 32 (1975): 623-631; Alan J. Sawyer, “Prospects for Integrated Pest Management of the Sea Lamprey (*Petromyzon marinus*),” *Canadian Journal of Fisheries and Aquatic Sciences* 37 (1980): 2083.

98 Tyler J. Buchinger, Michael J. Siefkes, Barbara S. Zielinski, Cory O. Brant, and Weiming Li, “Chemical Cues and Pheromones in the Sea Lamprey (*Petromyzon marinus*),” *Frontiers in Zoology* 12 (2015): 1-11; Skye D. Fissette, Tyler J. Buchinger, C. Michael Wagner, Nicholas S. Johnson, Anne M. Scott, and Weiming Li, “Progress Towards Integrating an Understanding of Chemical Ecology Into Sea Lamprey Control,” *Journal of Great Lakes Research* 47 (2021): 660-672; Lee H. Hanson, “Sterilization of Sea Lampreys (*Petromyzon marinus*) by Immersion in an Aqueous Solution of Bisazir,” *Canadian Journal of Fisheries and Aquatic Sciences* 38 (1981): 1285-1289; A.K. Lamsa, C.M. Rovainen, D.P. Kolenosky, and L.H. Hanson, “Sea Lamprey (*Petromyzon marinus*) Control – Where to From Here? Report of the SLIS Control Theory Task Force,” *Canadian Journal of Fisheries and Aquatic Sciences* 37 (1980): 2181-2189; Fred P. Meyer and Rosalie A. Schnick, “Sea Lamprey Control Techniques: Past, Present and Future,” *Journal of Great Lakes Research* 9 (1983): 354-358.

